# Recurrent central retinal artery occlusion as the initial manifestation of essential thrombocythemia: A case report

**DOI:** 10.1097/MD.0000000000048683

**Published:** 2026-05-08

**Authors:** Minyu Chen, Chi Du, Hongyan Lan, Jiahui Liu

**Affiliations:** a Ophthalmology Department, The Tenth Affiliated Hospital, Southern Medical University (Dongguan People’s Hospital), Dongguan, China; b Ophthalmology Department, Dongguan Qingxi Hospital, Dongguan, China.

**Keywords:** central retinal artery occlusion, essential thrombocythemia, paracentral acute middle maculopathy, thrombocytosis, transient monocular blindness

## Abstract

**Rationale:**

Essential thrombocythemia (ET) is a myeloproliferative neoplasm characterized by sustained thrombocytosis, which can lead to thrombotic complications including retinal artery occlusion. We report a rare case of recurrent central retinal artery occlusion (CRAO) as the initial manifestation of ET.

**Patient concerns:**

A 37-year-old female presented with recurrent transient monocular blindness in the right eye, progressing to persistent amaurosis. Ophthalmic examination revealed findings consistent with acute CRAO, accompanied by significantly elevated platelet counts. Emergency treatment for CRAO restored visual acuity from no-light-perception to 0.8 within 1 hour. Optical coherence tomography showed increased diffuse reflectance at the level of the inner nuclear layer consistent with paracentral acute middle maculopathy. Two recurrent CRAO episodes occurred over the following 2 years, with platelet counts persistently elevated.

**Diagnoses:**

Bone marrow biopsy revealed megakaryocytic hyperplasia, and genetic testing confirmed JAK2 mutation, establishing the diagnosis of ET.

**Interventions:**

The patient was treated with hydroxyurea and aspirin.

**Outcomes:**

Following treatment, the patient achieved normalization of platelet levels with no further episodes of amaurosis. However, discontinuation of medication led to recurrent thrombocytosis and a subsequent cerebral infarction.

**Lessons:**

This case highlights that recurrent CRAO in young patients should prompt investigation for underlying hematological disorders such as ET. Early diagnosis, appropriate cytoreductive and antiplatelet therapy, and long-term multidisciplinary follow-up are essential to prevent vision loss and life-threatening thrombotic complications.

## 1. Introduction

Essential thrombocythemia (ET) is a chronic myeloproliferative neoplasm characterized by clonal overproduction of platelets, carrying a long-term risk of progression to myelofibrosis and/or acute myeloid leukemia.^[1]^ While ET often presents asymptomatically (diagnosed incidentally via routine blood tests), it carries significant risks of thrombotic and hemorrhagic complications due to abnormal platelet function and hyperviscosity.^[2]^ It is especially noteworthy that thrombotic events, such as stroke and myocardial infarction, are the leading cause of mortality, occurring in 10% to 30% of ET patients.^[2]^

Ophthalmic manifestations of ET are rare, accounting for <5% of systemic complications, and most commonly include retinal vascular changes (e.g., retinal hemorrhage, venous occlusion).^[3,4]^ Central retinal artery occlusion (CRAO), an acute ophthalmic emergency causing rapid vision loss, is an extremely uncommon initial presentation of ET – with only a handful of cases reported in the literature and may precede other systemic thrombotic events.^[5,6]^

In this report, we describe a patient with recurrent CRAO as the initial manifestation of previously undiagnosed ET, who subsequently achieved complete visual recovery. This case underscores the importance of conducting a comprehensive systemic evaluation in young patients presenting with retinal vascular occlusions.

## 2. Case presentation

A 37-year-old female presented to our ophthalmology department in July 2018 with recurrent episodes of transient monocular blindness in her right eye. She described gradual visual obscuration lasting 5 to 7 seconds, followed by complete blindness for 40 to 50 seconds, with spontaneous resolution but recurred after 10 to 20 minutes. Thirty minutes before admission, her right eye developed persistent amaurosis. Her medical history included pregnancy-induced hypertension, hepatitis B, and a history of surgery and chemotherapy for a left facial malignancy. She had not undergone regular follow-up for any of these conditions.

**Figure 1.**
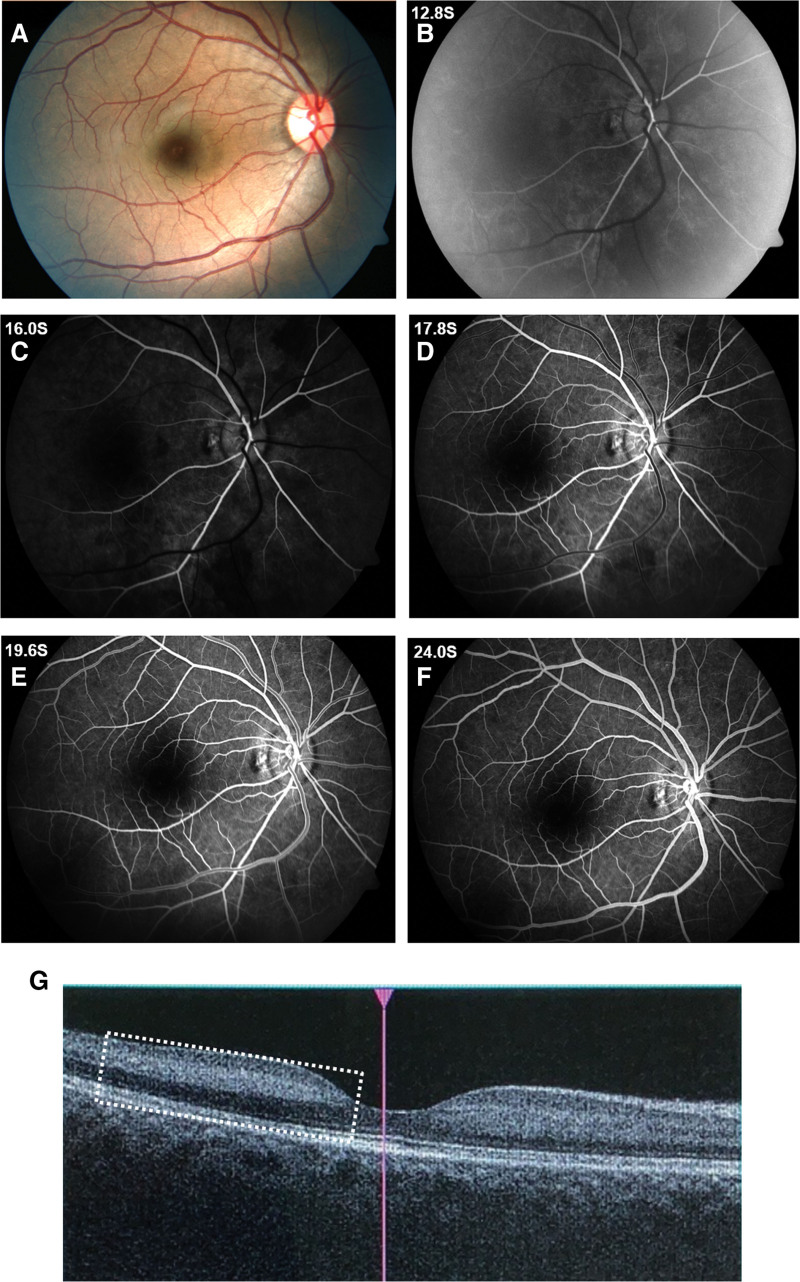
Multimodal imaging of the right eye. (A) Fundus photograph showing restoration of retinal arterial flow, but the arteries were narrowed, and the retina was mildly pale and edematous. (B–F) FFA revealed that the arterial phase began at 12.8 seconds, yet no obvious laminar flow was observed in the vein until 17.8 seconds. The vein remained incompletely filled at 19.6 seconds and did not fully enter the venous phase until 24 seconds, suggesting relatively slow retinal blood flow. (G) Macular OCT demonstrated mild inner retinal edema, with temporal hyperreflectivity spanning the inner nuclear to the outer plexiform layers, which is compatible with PAMM. FFA = fluorescein angiography, OCT = optical coherence tomography, PAMM = paracentral acute middle maculopathy.

**Figure 2.**
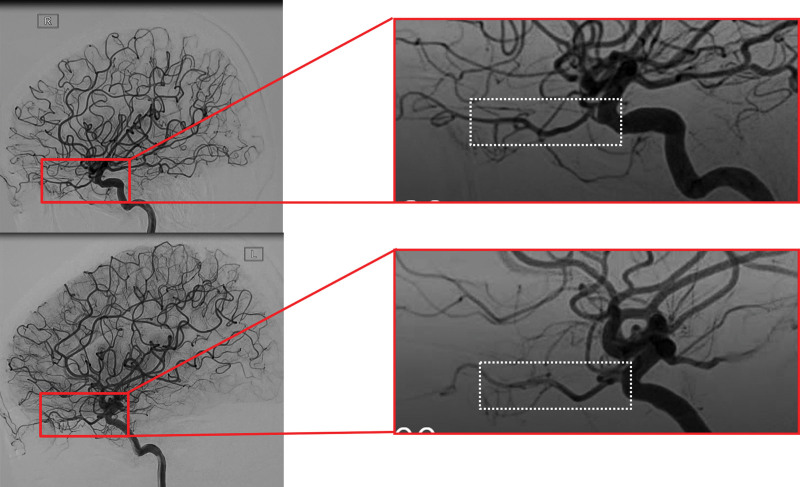
Digital subtraction angiography showing no significant stenosis but greater tortuosity and narrowing of the right ophthalmic artery compared to the left.

Ophthalmologic examination revealed visual acuity of no-light perception in the right eye, with a dilated pupil and absent direct light reflex. Fundus examination demonstrated diffuse retinal whitening in the posterior pole, a cherry-red spot, and marked retinal arterial narrowing without visible emboli. A diagnosis of acute CRAO was established. Emergency management was immediately initiated, including ocular massage, oxygen therapy, vasodilators, antiplatelet agents, and intraocular pressure-lowering treatment. Within 1 hour, her corrected visual acuity improved to 0.8 (−1.00DS/−1.25DC × 101°). Fundus reevaluation showed restoration of retinal arterial blood flow although the retina was less pale and edematous (Fig. 1A). Subsequent fluorescein angiography demonstrated markedly delayed retinal perfusion: the arterial phase began at 12.8 seconds (Fig. 1B), but laminar venous flow was not observed at 16 seconds (Fig. 1C) and did not appear until 17.8 seconds (Fig. 1D). Venous filling remained incomplete at 19.6 seconds (Fig. 1E), and full venous phase was not achieved until 24 seconds (Fig. 1F), collectively indicating sluggish retinal blood flow. Optical coherence tomography (OCT) revealed mild macular edema and hyper-reflective changes within the inner nuclear layer and inner plexiform layer consistent with paracentral acute middle maculopathy (PAMM; Fig. 1G). Initial laboratory workup identified elevated platelet counts (583 × 10^9^/L; normal range: 125–350 × 10^9^/L). Carotid ultrasound revealed increased resistance in the vertebral arteries. Computed tomography angiography suggested a suspected carotid-cavernous fistula, which was subsequently excluded by digital subtraction angiography. Digital subtraction angiography revealed no significant pathological stenosis of major intracranial vessels, but noted that the right ophthalmic artery was more tortuous and narrower than the left one (Fig. 2). The patient declined bone marrow biopsy to investigate the cause of thrombocytosis and was discharged from the hospital.

**Figure 3.**
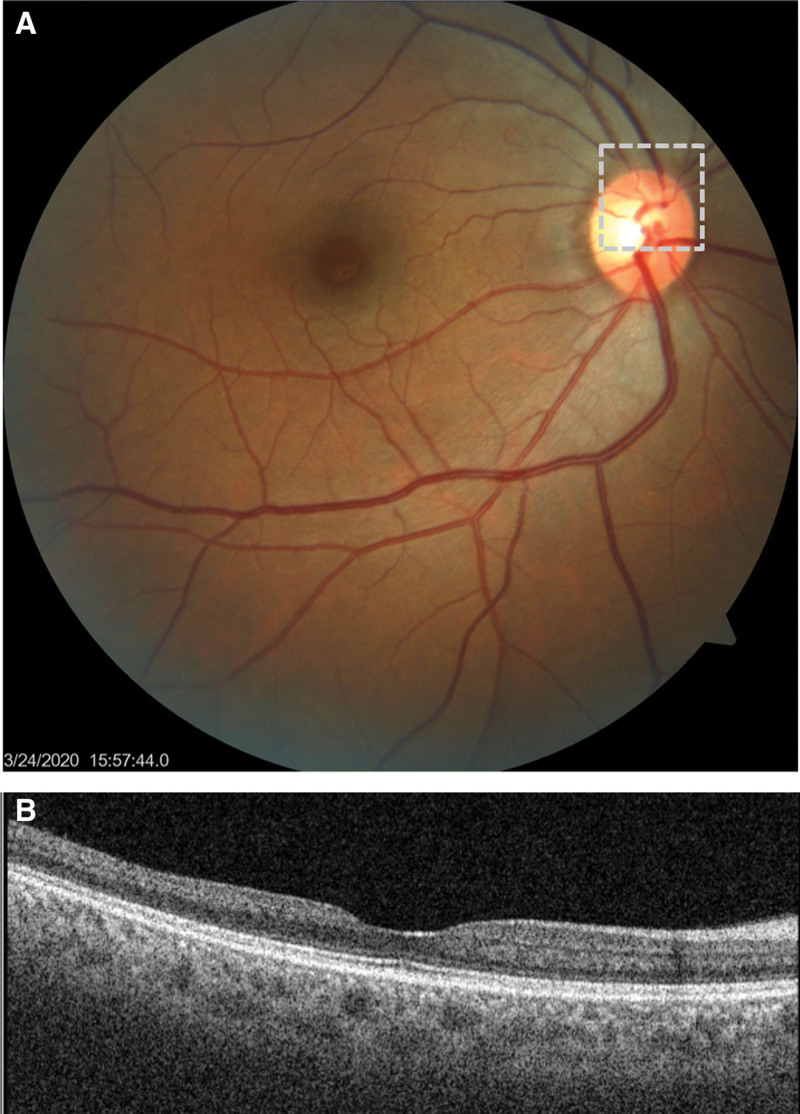
Fundus photograph and OCT images of the same patient at her third examination. (A) Fundus photograph showing an interruption of blood flow in the upper branch of the central retinal artery, which is presumably caused by a thrombus. (B) OCT showing progressive thinning of the retinal thickness in the right macular region. OCT = optical coherence tomography.

**Figure 4.**
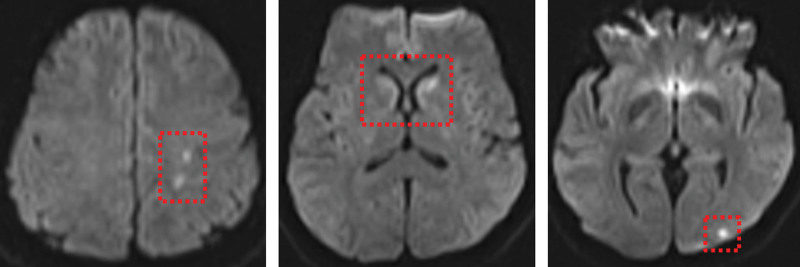
MRI showing scattered multiple abnormal signals in the left frontal-parietal-occipital lobes and bilateral caudate nuclei heads, suggestive of ischemic degenerative foci and cerebral infarction lesions. MRI = magnetic resonance imaging.

Similar CRAO episodes recurred in 2019 and 2020, each associated with elevated platelet counts (603 × 10^9^/L and 607 × 10^9^/L, respectively), with spontaneous visual recovery. During her third admission in 2020, a visible thrombus in the upper branch of the right central retinal artery was observed (Fig. 3A), and OCT showed progressive retinal thinning in the right eye (Fig. 3B). The patient subsequently consented to bone marrow biopsy, which demonstrated trilineage hyperplasia with prominent megakaryocytes. Genetic testing was positive for the JAK2 mutation and negative for the BCR-ABL fusion gene. Flow cytometry and karyotype analysis were normal, excluding other hematologic malignancies. Based on these examination results, the diagnosis of ET was confirmed. Hydroxyurea (0.5 g tid) for cytoreduction and aspirin (100 mg qd) for antiplatelet therapy were initiated. Platelet counts gradually normalized within 12 months, with no further amaurosis episodes during treatment adherence. Ophthalmic follow-up showed stable visual acuity, no recurrent CRAO, and no progression of retinal thinning. However, self-discontinuation of therapy during the COVID-19 pandemic led to recurrent thrombocytosis and a sudden left-sided weakness and slurred speech occurred in March 2022. Magnetic resonance imaging of the brain showed an acute left-basal-ganglia infarction (Fig. 4). Reinitiation of treatment stabilized her condition. As of December 2023 (last follow-up), the patient remains compliant with therapy: platelet count 230 × 10^9^/L, right eye visual acuity 0.8 corrected, no recurrent CRAO or neurological symptoms.

## 3. Discussion

CRAO typically affects older adults and is strongly associated with age-related vascular risk factors such as hypertension, diabetes, hyperlipidemia, and atherosclerosis. However, in young adults without conventional vascular risk factors, CRAO should raise suspicion for underlying hematological, inflammatory, or hypercoagulable conditions. Here we present a case of recurrent CRAO in a young patient who was eventually diagnosed with ET. The patient’s young age, recurrent presentation, and significant thrombocytosis were key indicators of an underlying systemic condition.

The patient’s ophthalmic manifestations, including retinal arterial narrowing and reduced retinal perfusion, align with ET-related hyperviscosity and thrombotic tendency. The recurrence of CRAO in this patient can be attributed to 4 key factors: thrombocytosis-induced hyperviscosity and platelet hyperactivity: ET is characterized by clonal platelet overproduction, with platelets exhibiting increased adhesion, aggregation, and prothrombotic activity.^[1]^ In this patient, persistent thrombocytosis led to increased blood viscosity (reducing retinal arterial flow) and enhanced platelet aggregation, creating a prothrombotic microenvironment that predisposed to CRAO. This is supported by studies showing that platelets affect arteriolar endothelial function through pathways such as P-selectin,^[7]^ and higher platelet count was associated with narrower arteriolar caliber;^[8,9]^ JAK2 mutation-related prothrombotic microenvironment: JAK2, a member of the non-receptor tyrosine kinase family, mediates the proliferation of hematopoietic cells through its activation by cytokines, including erythropoietin and thrombopoietin.^[10]^ About 50% to 60% of ET patients harbor a JAK2 variant (most commonly JAK2 V617F).^[11]^ The JAK2 mutation induces a pro-inflammatory state in myeloid cells, notably by enhancing neutrophil adhesion and the formation of neutrophil extracellular traps, which directly promote thrombus formation.^[12]^ It also causes qualitative abnormalities in red blood cells and platelets, increasing their rigidity, adhesion, and the release of pro-coagulant microvesicles, thereby elevating blood viscosity and thrombin generation. Furthermore, the mutation can directly activate the vascular endothelium, leading to increased expression of pro-adhesive and pro-coagulant proteins like P-selectin and von Willebrand factor, which creates a hypercoagulable environment at the vessel wall. The JAK2 mutation is a key determinant of thrombosis and is integral to risk stratification in ET.^[13]^ Anatomical vulnerability: the pathophysiological mechanisms of right ophthalmic artery tortuosity, which increases vascular resistance and fosters flow stagnation, acted in concert with the prothrombotic milieu of ET to jointly facilitate thrombus formation. Chronic retinal ischemia: cumulative retinal ischemic injury from repeated occlusions led to progressive retinal thinning, compromising retinal tissue resilience and increasing susceptibility to recurrent retinal arterial occlusion.

Notably, this patient experienced significant visual recovery following acute CRAO episodes, which contrasts with the typically poor visual prognosis in ET-associated CRAO.^[5,6]^ This discrepancy may be partially explained by the degree of thrombocytosis. Reported platelet counts in ET patients with poor visual outcomes following CRAO were substantially higher (e.g., 896 × 10^9^/L and >1000 × 10^9^/L, respectively) than in our patient (583 × 10^9^/L).^[5,6]^ Higher platelet counts may contribute to a greater burden of microthrombi and a more prothrombotic state, potentially leading to more sustained or complete vascular occlusion. The core pathological issue of ET is a predisposition to microthrombosis driven by elevated platelet counts. These microthrombi are prone to recurrent embolization within the retinal arterial system. However, due to their small size or activation of the endogenous fibrinolytic system, these embolic events may undergo spontaneous resolution without causing sustained and complete occlusion, allowing retinal blood flow to be reestablished before the development of panretinal infarction.

PAMM is thought to represent a reversible ischemic change in the deep capillary plexus, triggered by fluctuations in perfusion pressure.^[14]^ Recent studies suggest that PAMM represents a vasoreactive compensatory response that regulates vascular resistance during fluctuations in perfusion pressure, thereby maintaining tissue perfusion.^[15–17]^ In this patient, the phenomenon of “spontaneous resolution” essentially reflects the dissolution of microthrombi (e.g., via activation of the fibrinolytic system), leading to the relief of deep capillary plexus ischemia. PAMM, observed on OCT, represents an objective imaging marker of her recurrent episodes of incomplete CRAO or transient retinal arterial hypoperfusion. This explains the remarkable and complete recovery of her visual acuity following clinically apparent CRAO-like events. Each ischemic episode did not result in irreversible damage to neural elements such as retinal ganglion cells, thereby allowing her to avoid the irreversible panretinal infarction. Thus, visual acuity remained fully recoverable despite recurrent ischemic attacks. This aligns with research suggesting that PAMM may serve as a potential adaptive mechanism to microvascular occlusion, contributing to better visual outcomes in select CRAO cases.^[18–20]^

Multidisciplinary collaboration among ophthalmologists, hematologists, and neurologists is essential to secure early diagnosis, institute appropriate therapy, and monitor compliance. Lifelong cytoreductive therapy (hydroxyurea or, in resistant cases, ruxolitinib) combined with low-dose aspirin is currently the evidence-based standard for high-risk ET. Compliance with cytoreductive and antiplatelet therapy is vital to prevent recurrent thrombotic events, as demonstrated by the patient’s cerebral infarction following treatment discontinuation.

## 4. Conclusion

Recurrent CRAO in young patients should raise clinical suspicion for underlying hematological disorders such as ET. Early diagnosis, prompt treatment, and coordinated long-term follow-up are key to preventing thrombotic complications and preserving visual and neurological function. Patient education and regular monitoring are imperative to ensure treatment adherence and improve outcomes.

## Author contributions

**Conceptualization:** Minyu Chen, Jiahui Liu.

**Data curation:** Chi Du, Hongyan Lan.

**Supervision:** Jiahui Liu.

**Writing – original draft:** Minyu Chen.

**Writing – review & editing:** Chi Du, Hongyan Lan, Jiahui Liu.
